# Experiences and responses of second victims of patient safety incidents in Korea: a qualitative study

**DOI:** 10.1186/s12913-019-3936-1

**Published:** 2019-02-06

**Authors:** Won Lee, Jeehee Pyo, Seung Gyeong Jang, Ji Eun Choi, Minsu Ock

**Affiliations:** 1 Asian Institute for Bioethics and Health Law, Seoul, Republic of Korea; 2 Department of Medical Humanities and Social Sciences, Division of Medical Law and Bioethics, Yonsei University College of Medicine, Seoul, Republic of Korea; 3 Department of Preventive Medicine, Ulsan University Hospital, University of Ulsan College of Medicine, 877, Bangeojinsunhwando-ro, Dong-gu, Ulsan, 44055 Republic of Korea; 4 Doctoral Program in Medical Law and Ethics, Yonsei University, Seoul, Republic of Korea; 5 Office of Research Planning and Coordination Department, National Evidence-based Healthcare Collaborating Agency, Seoul, Republic of Korea

**Keywords:** Patient safety, Second victims, Medical litigation, Republic of Korea, Qualitative research, Grounded theory, Emotional distress

## Abstract

**Background:**

Healthcare professionals who experience trauma due to patient safety incidents can be considered second victims, and they also suffer from various difficulties. In order to support second victims, it is necessary to determine the circumstances of the incidents in question, along with the symptoms that the victims are experiencing and the support they require. A qualitative study on healthcare professionals of various occupations, such as physicians and nurses working in Korea, was conducted, and the experiences and response methods and processes of second victims were examined.

**Methods:**

In-depth interviews were conducted with 16 healthcare professionals (six physicians, eight nurses, and two pharmacists) who had experienced a patient safety incident. All interviews were recorded and transcribed, and the data analysis was conducted in accordance with Strauss and Corbin’s grounded theory. Both open coding and axial coding were performed. Consolidated criteria for reporting qualitative research (COREQ) were applied in this study.

**Results:**

The results of the open coding demonstrated that the experiences of second victims can be categorized into “the reactions of the first victim and surrounding people after the incident,” “Influence of factors aside from the incident,” “the initial complex responses of the participants to the incident,” “open discussion of the incident,” “the culture in medical institutions regarding early-stage incident response,” “the coping responses of the participants after incidents,” and “living with the incident.” Then, the seven categories in the open coding stage were rearranged according to the paradigm model, and the reaction process of the second victims was analyzed through process analysis, being divided into the “entanglement stage,” “agitating stage,” “struggling stage,” “managing stage,” and “indurating stage.”

**Conclusions:**

This research is significant because it provides a comprehensive understanding of second victims’ experiences in the eastern region of Korea, by obtaining data using a qualitative research method. The findings of the study also highlight the five stages of the second victim response process, and can be used to design a specialized second victim support program in Korea.

**Electronic supplementary material:**

The online version of this article (10.1186/s12913-019-3936-1) contains supplementary material, which is available to authorized users.

## Background

Patient safety incidents (PSIs) can cause physical and mental damage to patients and their caregivers [1, 2]. However, the trauma caused by such accidents is not limited to the patient and his/her caregiver. The healthcare professionals who are involved in the incident, the so-called second victims, can also experience distress, such as guilt, anger, frustration, psychological stress, and fear, as well as physical symptoms such as fatigue, insomnia, and aberrant behaviors [3–5]. In addition, apart from emotional and behavioral changes, such healthcare professionals have been found to contemplate switching careers [6], and have decreased job satisfaction [7]. Experiencing a PSI can have a lasting effect on every healthcare professional, regardless of their occupation, career length, or sex [6].

Estimates of the incidence rate of PSIs suggest that numerous healthcare professionals may be the second victims at some point in their careers [8, 9]. However, at present, the support provided by medical institutions and peers to second victims of PSIs is insufficient [7, 10, 11]. Appropriate support and assistance for such second victims may alleviate their emotional stress, but if negative attitudes exist in their medical institutions and among colleagues regarding such incidents, the second victims’ emotional burdens could be aggravated [6, 7]. In particular, it is essential to provide specific and adequate endorsement and support for second victims, who may be continuing to treat patients while experiencing emotional distress as a result of the incident, which in turn increases the risk of another such incident occurring [12].

In order to support second victims, it is necessary determine the circumstances of the incidents in question, along with the symptoms that the victims are experiencing and the support they require. In addition, an in-depth investigation of the second victims’ response processes is needed. Most previous studies on second victims have focused on their symptoms, but research on specific response steps for such individuals is relatively scarce [4]. Although Scott et al. [6] categorized the general recovery of second victims into six main phases, additional studies that focus on the response phase are needed to generalize the existing studies’ findings. In Western countries, various researches, such as the qualitative research and the development of the support programs related to the second victims, have been conducted, but to our best knowledge, there is a lack of research in Asian region, except one study [13]. Considering the possible influences of cultural differences on patient safety, it is imperative to examine the effects of cultural context on second victims’ responses.

In this study, we attempted to analyze the response processes of second victims in the Republic of Korea (hereinafter “Korea”). Specifically, a qualitative study on healthcare professionals of various occupations, such as physicians and nurses working in Korea, was conducted, and the experiences and response methods and processes of second victims were examined.

## Method

This was a qualitative study in which in-depth interviews were conducted with healthcare professionals who had experienced a PSI, and data were collected and analyzed using the grounded theory method. Consolidated criteria for reporting qualitative research (COREQ) were applied in this study [14].

### Research team

The research team comprised five researchers who had experience in qualitative research. Further, all five had specialized in the field of healthcare and patient safety research, as one was a physician, three were nurses, and one had a degree in counseling.

### Participants and data collection

The study participants were healthcare professionals who had experienced PSIs. Convenience sampling was applied in this study, because it is difficult to openly recruit healthcare professionals who have experienced PSIs. There are still views that PSIs occur due to lack of expertise and hence people do not overtly discuss their experience of PSIs in Korea. Initially, the researchers contacted potential participants individually and then through those who agreed to participate, snowballing sampling was employed to recruit additional participants.

The participants were also selected using the theoretical sampling and purposive sampling methods. In other words, experiences of and countermeasures taken to address PSIs, with consideration of type of occupation, type of institution, job status, and the severity of the incident, were regarded when selecting participants. Additionally, participants from the tertiary hospital were recruited, because the strategy and policy for the medical professionals may differ depending on the type of institution, and it is necessary to comprehend the current situation and develop management plans as tertiary hospitals are highly affected by the laws and policies related to patient safety. In particular, during the initial recruitment, physicians who were specialists and professors were prioritized and consequently interviewed. However, in the course of the research, as it was necessary to confirm the experiences of residents who practice medicine in hospitals under the supervision of an attending physician, further residents were recruited and interviewed. No individual refused to participate in the study. In the final study, there were a total of 16 participants: six physicians, eight nurses, and two pharmacists. The main characteristics of the study participants are shown in Table 1. The types of PSIs and degree of harm due to PSIs [15] are also presented in Table 1.

**Table 1:** Socio-demographic characteristics of the study participants and the type of patient safety incidents

	Sex/Age	Occupation	Department	Work experience (years)	Patient safety incidents	
					Incident Type	Degree of harm
1	F/20s	Pharmacist	Pharmacy	7	Behavior (Noncompliant/Uncooperative/Obstructive)	None
					Medication/IV Fluid	None
2	F/30s	Pharmacist	Pharmacy	9	Medication/IV Fluid	None
					Medication/IV Fluid	None
3	F/20s	Nurse	Gynecology	3	Clinical Process/Procedure	None
					Documentation	None
4	F/30s	Nurse	Emergency medicine	9	Patient Accidents (Falls)	Mild
					Patient Accidents (Falls)	None
					Medication/IV Fluid	None
5	F/20s	Nurse	Neurology	6	Clinical Process/Procedure	Moderate
6	M/30s	Physician	Orthopedics	6	Clinical Process/Procedure	Severe
					Resources/Organizational Management	Death
7	F/30s	Physician	Emergency medicine	8	Clinical Process/Procedure	Death
8	F/30s	Nurse	Surgery	8	Blood/Blood Products	None
					Clinical Process/Procedure	Death
					Medication/IV Fluid	None
9	F/40s	Physician	Pulmonology	21	Clinical Process/Procedure	Death
					Clinical Process/Procedure	None
10	F/30s	Physician	Infectious disease	9	Clinical Process/Procedure	Severe
11	M/40s	Physician	Cardiology	14	Clinical Process/Procedure	Severe
12	F/20s	Nurse	Neurosurgery	6	Patient Accidents (Falls)	None
13	F/40s	Nurse	Pediatrics	24	Clinical Process/Procedure	Death
14	F/30s	Nurse	Pediatrics	10	Clinical Process/Procedure	Death
					Medication/IV Fluid	None
15	F/30s	Nurse	Nursing	10	Behavior (Intended Self Harm/Suicide)	Death
					Behavior (Intended Self Harm/Suicide)	None
16	M/30s	Physician	Pediatrics	8	Clinical Process/Procedure	Death

Interviews were conducted from July 2017 to March 2018, until the content reached saturation level. The interviews were semi-structured, and focused on the experiences with PSIs, mental and physical responses immediately after the incidents, support received during the response process, reactions of peers or superiors, and so on (Additional file 1). They lasted for an average of one hour, and further telephone interviews were conducted if additional inquiry and data collection were required.

### Data analysis

The data analysis was conducted in accordance with Strauss and Corbin’s grounded theory [16], which allowed us to obtain a comprehensive understanding of the participants’ response experiences after the occurrence of PSIs, the situations that arise as a result, specific responses, and coping processes; further, we could categorize these into a respective series of steps. In qualitative studies, especially those using grounded theory, data analysis proceeds simultaneously with data collection, and participants are additionally recruited for establishing the theory. All interviews were recorded and transcribed. One researcher led the first analysis and the other four researchers performed the second review. In addition, all the researchers collectively reviewed the parts that needed consensus and discussion.

**Fig. 1:**
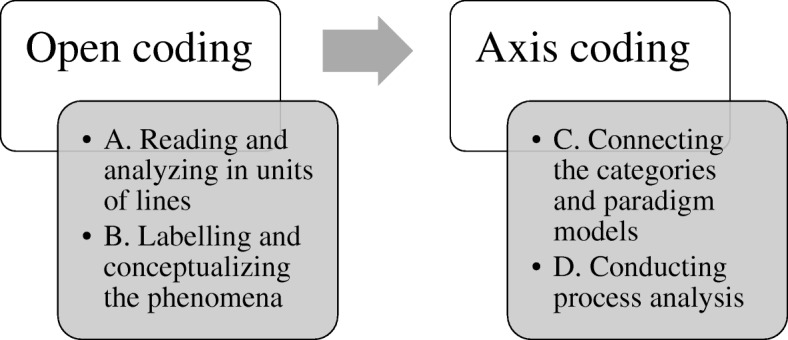
Process of open coding and axial coding

Both open coding and axial coding were performed. In the open coding, initially, the transcribed data were read and analyzed in units of lines, with the aim of gaining an understanding of the events experienced and the response processes performed by the participants (A, Fig. 1). After that, phenomena shown in the data were labeled, and the incidents were conceptualized and categorized. Through this analysis, concepts, categories, and subcategories which are commonly used in grounded theory, were derived (B, Fig. 1). In axial coding, paradigm models were used to connect the categories derived from the open coding process. Paradigm models consisted of causal conditions, central phenomena, contextual and interventional conditions, action/interaction strategies, and results (C, Fig. 1). Additionally, a process analysis was conducted to track changes in the situation over time by searching for continuous connections between categories and subcategories (D, Fig. 1). In other words, the research team derived the steps of response for a second victim based on repeated discussions.

### Research validity assessment

To assess the validity of the qualitative research, the applicability, consistency, and neutrality examinations recommended by Guba and Lincoln were applied [17]. Two participants, who were included in the 16 interviewed and shared abundant experience in the interview, agreed to assist in a confirmation process voluntarily, in which they were asked to confirm the research results, which served to increase the realistic value. Moreover, to validate the research feasibility, the results were presented to a physician and a nurse who were non-participants in the research and who had PSI experience; these individuals were able to compare the results with their own experiences and response processes regarding PSIs. Further, to increase consistency and ensure neutrality, all five researchers were careful that the researchers’ specific experiences did not affect the analysis in the process of data analysis. Moreover, we continued to share the opinions of the researchers and held regular meetings to maintain neutrality in the analysis process.

### Ethics

This study was performed after receiving approval from the institutional review board (IRB) of Ulsan University Hospital (IRB number: UUH 2017–05-037), and conducted in accordance with the Declaration of Helsinki. Before the interviews, a researcher informed the participants of the purpose of the study, that their privacy would be protected, that they could withdraw participation at any time without penalty, and that the interviews would be recorded; after this, all participants provided written informed consent. The participants were also notified that all of the content and research data would not be used for any purpose other than the present research, and would be destroyed at the end of the research.

## Results

### Open coding

From the original data, 821 concepts were identified via open coding, comprising seven categories and 14 subcategories (see Table 2).

**Table 2:** Open coding results

Category	Sub-category	Main concept
1. The reactions of the first victim and surrounding people after the incident	1–1 Caregiver’s response after an incident	1–1-1 Understanding and accepting type 1–1-2 Non-responsive type 1–1-3 Emotional-reaction type 1–1-4 Behavior-expressing type
	1–2 Fellow healthcare professionals’ responses after an incident	1–2-1 Consoling and sympathetic type 1–2-2 Blaming and reprimanding type 1–2-3 Non-responsive and impassive type 1–2-4 Scapegoat type
2. Influence of factors aside from the incident	2–1 Influence of work-related factors	
	2–2 Influence of healthcare professionals’ characteristics	
3. The initial complex responses of the participants to the incident	3–1 Emotional response to the incident	3–1-1 Emotional response to patients and caregivers due to the incident 3–1-2 Self-focused emotional response due to the incident 3–1-3 Emotional response to fellow healthcare professionals and institutions
	3–2 Behavioral responses to the incident	3–2-1 Behavioral responses to patients and caregivers due to the incident 3–2-2 Self-focused behavioral responses as a result of the incident 3–2-3 Behavioral responses to fellow healthcare professionals and institutions
4. Open discussion of the incident	4–1 Atmosphere that encourages the disclosure of incidents	4–1-1 Atmosphere of addressing the incident openly 4–1-2 Atmosphere of not addressing the incident openly
	4–2 The disclosing of an incident depends on the degree of severity involved	4–2-1 Significant severity 4–2-2 Mediocre severity 4–2-3 Difficult to disclose incidents regardless of severity
	4–3 Disclosure is based on patients’ awareness of incidents	4–3-1 Disclosing incidents when patients became aware of the incidents 4–3-2 Avoiding disclosure of incidents when patients do not notice the incidents
5. The culture in medical institutions regarding early-stage incident response	5–1 Positive culture	
	5–2 Negative culture	
6. The coping responses of the participants after incidents	6–1. Work-level coping response to prevent recurrence of incidents	6–1-1 Proactive coping response 6–1-2 Passive coping response
	6–2. Personal efforts to resolve psychological difficulties	6–2-1 Endeavors to overcome the incident independently 6–2-2 Endeavors to overcome the incident with the help of others
7. Living with the incident	7–1. Trauma that is less severe but still present	7–1-1 Improved 7–1-2 Engraved in memory 7–1-3 Affects work
	7–2. Assistance in accepting the trauma	7–2-1 Emotional assistance provided to healthcare professionals 7–2-2 Administrative assistance provided to healthcare professionals

#### The reactions of the first victim and surrounding people after the incident

In the study, the responses of patients, caregivers, and fellow healthcare professionals to a PSI were found to have an influence on participants’ initial responses to the PSI. The participants reported experiencing various patient and caregiver reactions after their PSI: some patients and caregivers demonstrated no particular response to the incident, but others expressed anger and/or filed lawsuits.

*“I remember one thing […] the patient told me that I was not a qualified nurse… I honestly do not remember the other comments.” (Participant 12).*

*“Because (the patient) died that day, the caregiver believed it was the fault of the healthcare professionals; as a result, I am now facing trial.” (Participant 13).*

One participant met with the caregiver every day after the PSI and explained the progress of the patient; this eventually led to the caregiver gaining an understanding of the participant’s approach and mindset. Participants also claimed that the level of trust between the healthcare professionals and the patient and the caregiver (if applicable) had an impact on the response to and resolution of PSIs.

*“I was on good terms with the caregiver. So I did not have to worry about being blamed by the caregiver…” (Participant 9).*

Fellow healthcare professionals’ process of recognizing and responding to PSIs also influenced the participants’ acceptance of and responses to the incidents. After a PSI, some colleagues shared their previous mistakes and expressed sympathy with the participants, but others were angry and publicly criticized the participants during shifts and in mortality and morbidity conferences. Furthermore, others had impassive responses, neither criticizing nor consoling the participants, or imputed the responsibility of the incident to another individual or department.

*“While I was sleeping in my home after work, he/she called me and angrily screamed at me […]: ‘what the hell have you done?’” (Participant 8).*

*“There was a debate. […] I kept telling them what really happened, but the trauma team kept saying that we were responsible.” (Participant 7).*

#### Influence of factors aside from the incident

Work-related factors and the characteristics of healthcare professionals also affected the response process after PSIs. Highly experienced participants claimed that the frequency of such incidents had decreased as they gained experience. Some of these participants said they experienced pressure to avoid mistakes due to their experience, but others said they felt less pressurized. Additionally, some reported that, due to their heavy workloads, they did not have time to think about the incident itself or respond to the patient and/or caregiver.

*“If I were to be responsible for such an incident, I would be asked something like ‘you are too experienced to make such mistakes, so how did this happen?’.” (Participant 3).*

The participants, aside from those who were inherently prone to feeling less stress, also mentioned that characteristics such as perfectionism and timid personality affect the response process to such incidents.

*“I have a bit of a perfectionist personality… honestly… I haven’t had any problems other than that incident. So… yeah, my perfectionism helps.” (Participant 11).*

#### The initial complex responses of the participants to the incident

The participants experienced various emotional reactions to their PSIs, such as embarrassment, guilt, and depression, as well as behavioral changes such as insomnia, avoidance, and consideration of a career change. After the incident, they experienced embarrassment and fear regarding the fact that the incident occurred, and feelings of remorse and guilt towards the patients and their caregivers. In addition, they experienced complex emotional reactions such as a sense of shame for making a mistake and remorse for damaging trust in and the reputations of fellow healthcare professionals and institutions.

*“At first, it was too scary to think about what had happened to the patient.” (Participant 3).*

*“In the hospice room, the patient took his last breath. His guardian was furious, of course, because she was his wife… I could feel what she was going through… I was very shocked.” (Participant 7).*

In particular, the participants’ complex emotional responses had an extremely pronounced impact on their behavioral changes. They struggled to avoid showing guilt or fear to the patients and caregivers.

*“I have not been in the elevator for a while, because I could meet… I could meet the caregiver there… […] So, I have not been in the elevator for about a month…” (Participant 10).*

Additionally, after a PSI, when participants were required to perform the same or similar work, they experienced symptoms such as tremors in their hands and headaches; consequently, they had difficulty executing their work, which led to insomnia and pain and, eventually, consideration of resignation or a career change. Additionally, their relationships with fellow healthcare professionals involved in the incident became strained, rather than a source of mutual support.

*“At that time, I tried to get a lot of certifications because I was thinking about finding a new job… I thought about quitting… I had the time to think about what to do, and from April I took a lot of TOEIC exams. I took TEPS and TOEIC, all of them.” (Participant 5).*

#### Open discussion of the incident

The atmosphere in the participants’ medical institutions regarding discussing PSIs affected how the participants responded to the PSIs. Some medical institutions openly discussed and addressed all PSIs, but others did not discuss such cases. In addition, in some institutions, the degree of severity of an incident influenced whether the incident was discussed; in some institutions only severe cases were discussed, while in others PSIs were discussed regardless of the type or severity of the risk.

*“We discuss all incidents, because we have all become cautious. If you write about it (an incident) in a report, it is shared during handover, and then all the ward nurses know. We are just trying to be careful.” (Participant 3).*

*“I didn’t explain. I didn’t tell the patient about it (the incident), even though explaining it, immediately saying ‘sorry,’ is the correct approach. The physician (who was in charge of the patient’s care) said that the mistake had not harmed the patient, and forget about it.” (Participant 8).*

Moreover, in some institutions, disclosure of a PSI differed based on whether the patient was aware of the incident. If the patient was aware of the incident, the case was discussed with them, and if not, the information was withheld.

*“The only case we can talk about is when we administer the wrong medicine… (You don’t mention the cases that go unnoticed, right?) Yes, we tend not to discuss those.” (Participant 1).*

*“Some caregivers and patients do not notice until we tell them, and sometimes we do not specifically tell them.” (Participant 8).*

#### The culture in medical institutions regarding early-stage incident response

The culture in their medical institutions influenced the participants’ responses to PSIs. In some institutions, rather than receiving accusations or reprimands after an incident, a supportive and positive culture was present, with procedures such as the seniors meeting and discussing the incident and colleagues providing assistance. On the other hand, other institutions had a negative culture, with measures such as conducting investigations to find the party responsible for the incident, with those responsible being interrogated by seniors and colleagues and being stigmatized.

*“Anyway, I think a newbie is a newbie and mistakes are bound to happen. I guess it would be better if mistakes are discovered before any harm is done, but I think it’s very wrong to criticize and put all of the responsibility on a newbie just because that person has made a mistake. The seniority system exists due to this, with those higher in the system acting as supervisors.” (Participant 8).*

*“If you acquire a reputation (of making mistakes), then people think of you as an incapable person… whenever you do a night shift or something, people blame you for not completing work or stuff… this kind of pressure becomes very burdensome…” (Participant 2).*

#### The coping responses of the participants after incidents

After incidents, the participants endeavored to overcome their experiences by improving their work. They particularly attempted to prevent a recurrence of the incident, mainly through proactive management, such as through habitual additional checks; meanwhile, others engaged in passive management, such as by avoiding performing the medical treatment that caused the problem.

*“Of course, I care for (patients) more meticulously now, like monitoring their breathing, for instance… I try to not to be excessive, but… I take better care of them now. I try to check every detail.” (Participant 5).*

The participants sought to overcome their PSI experiences by themselves or with the help of others, such as colleagues or friends, aiming to resolve the psychological difficulties associated with the response process and those encountered at a later stage. However, one participant mentioned that he/she was left to handle his/her own emotions and overcome the incident alone; additionally, discussing the incident with others served as a reminder of what had happened. Others who overcame the incident with the help of colleagues or friends mostly discussed the incident with colleagues from the same hospital or healthcare professionals, as non-healthcare professionals could not comprehend the situation or sympathize based on similar experiences.

*“I have a lot of discussions with friends who I practiced with and with friends who I attended school with.” (Participant 6).*

#### Living with the incident

The psychological difficulties and physical symptoms that occurred immediately after the PSIs improved over time, but remained engraved in the participants’ memories. In fact, even mentioning the incident caused some of them to cry, feel distressed, and/or feel resentful. Some participants mentioned experiencing flashbacks of the incident if a similar situation arose, and the reactions had experienced, such as verbal assaults, were still painful memories. Moreover, they experienced difficulties performing medical practices or adopted a defensive work attitude when they encountered situations related to the incident.

*“I know it’s an inevitable process because I am still learning, but now […], I guess I have gained experience because of it, but it has also become traumatic.” (Participant 9).*

*“I can no longer do it (the medical practice in question) since the incident. I found it easy before… […] now, performing the test is difficult for me.” (Participant 16).*

The participants reported the need for emotional and administrative support to help similar individuals overcome the psychological difficulties experienced as a result of a PSI. Psychological counseling was particularly suggested, as PSIs can cause post-traumatic stress disorder in healthcare professionals. They also mentioned that they were not aware of how to appropriately express feelings of regret and remorse, how to respond after an incident, and more. Moreover, they mentioned that administrative support should systematically address such incidents and discuss countermeasures.

*“I want to talk about the psychological trauma experienced by healthcare professionals […], even if I don’t have an opportunity to regularly share what I have been going through and console people who have had similar experiences, I think even if I only had occasional opportunities it would be really good.” (Participant 7).*

### Axial coding

#### Category analysis

**Fig. 2:**
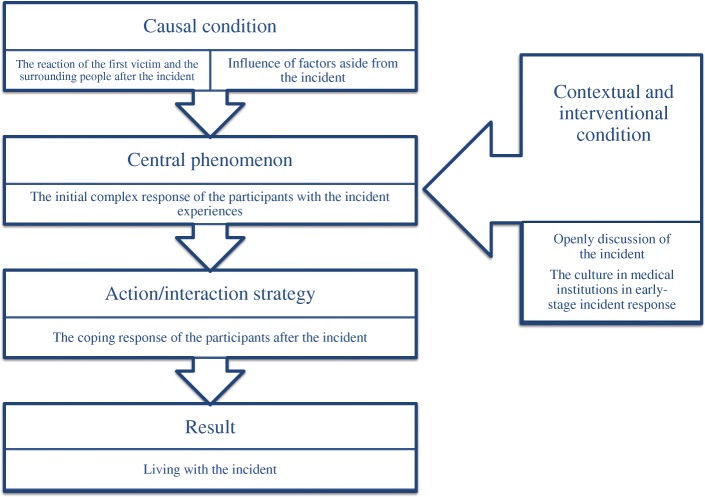
Paradigm model for axial coding

Figure 2 shows the rearrangement in the paradigm model of the seven categories that were analyzed in the open coding process. In this study, the central phenomenon was “the initial complex responses of the participants to the incident.” The reason for setting the central phenomenon as “the initial complex responses of the participants to the incident” is that participants showed complex reactions such as emotional as well as behavioral changes in the initial coping stage after the incident. The participants’ emotional reactions included remorse and guilt towards patients and caregivers, fellow healthcare professionals, and themselves after their PSI. They also avoided the parties involved, considered finding a new job or resigning, and experienced symptoms of somatization. The conditions that caused the experience were “the reactions of the first victim and surrounding people after the incident” and “the influence of factors aside from the incident.” As they occurred before the central phenomenon and it was, therefore, appropriate to presume them to be causes of the phenomenon. The categories of “open discussion of the incident” and “the culture in medical institutions regarding early-stage incident response” were decided to be contextual and interventional conditions, as they affected “the coping responses of the participants after incidents,” which were action/interaction strategies. The participants’ efforts to prevent a recurrence of the incidents and resolve their psychological difficulties were considered to be methods of addressing their initial responses, and were also determined to be action/interaction strategies. “Living with the incident” was postulated to be a result. Participants mentioned that the trauma caused by PSI gradually faded, but did not disappear, and that psychological and administrative assistance was needed to heal the wound.

#### Process analysis

**Fig. 3:**
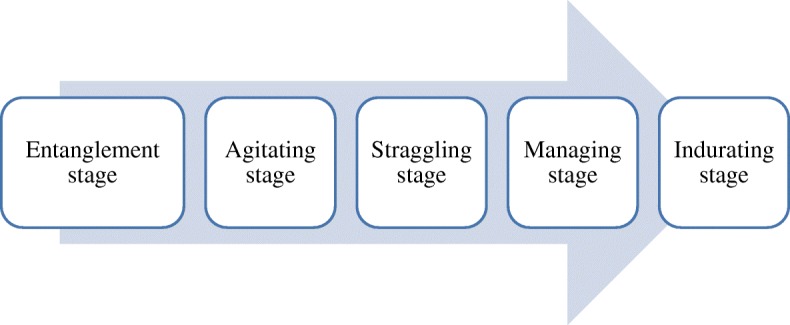
Response stages of second victims

Figure 3 shows the results of the step-by-step analysis of the participants’ responses to the PSIs. Their PSI experiences could be summarized into the “entanglement stage,” “agitating stage,” “struggling stage,” “managing stage,” and “indurating stage.” In the “entanglement stage,” they were overwhelmed with the incidents for which a PSI occurred and efforts were made to address and respond to the incident, such as by minimizing possible risks to the patient, responding to the patient and caregiver, and discussing the incident with colleagues. After the entanglement stage, the participants were distressed by the PSI and showed emotional, behavioral, and physical symptoms and we named it “agitating stage.” Due to their heavy workloads, many participants did not have the time to reflect on their feelings and conditions; moreover, some were disturbed by their emotional difficulties, avoided performing similar work, and considered finding a new job or resigning due to the reactions of patients and caregivers and peer medical staff. Many avoided patients, caregivers, and fellow healthcare professionals due to feelings such as remorse, guilt, disgust, and grudge. They also experienced fear or developed somatization symptoms when performing the same or similar tasks which had caused the incident. Of these difficulties, the worst was agonizing over their career, which led them to consider finding a new job or resign. However, the participants generally felt that they were duty-bound to overcome the PSIs and endeavored to resolve their difficulties. Researchers called this stage the “struggling stage.” To achieve this, they engaged in hobbies and talked with friends, colleagues, and family. After the “struggling stage,” in the “managing stage,” participants sought to overcome the PSI, followed by self-assessment and management of the impact of the incident. Thus, they made concerted efforts to surmount the incident and steadily regulated the influence of the PSIs. In particular, psychological difficulties and physical symptoms improved immensely over time. However, regardless of the efforts invested during the process, the PSI remained a significant wound for them, but seemed to be stagnant, as its painful effect had reduced. The researchers named this stage the “indurating stage,” focusing on memorable experience and dullness.

## Discussion

This research involved a qualitative study of Korea-based healthcare professionals across several occupations, such as physicians and nurses, with the aim of examining the experiences and response methods of second victims to PSIs. In-depth interviews with 16 healthcare professionals were performed, followed by open coding and axial coding, which were conducted in accordance with grounded theory. A total of seven categories and 15 subcategories were derived through open coding. The response process of the second victims was analyzed through process analysis, being divided into five stages as follows: “entanglement stage,” “agitating stage,” “struggling stage,” “managing stage,” and “indurating stage.”

As there is insufficient literature specifically exploring second victims’ response processes to PSIs, the findings of this study are expected to assist in the development of programs that specifically aim to address the psychological problems of second victims. Scott et al. classified the general recovery of second victims into six stages: (1) chaos and accident response, (2) intrusive reflections, (3) restoring personal integrity, (4) enduring the inquisition, (5) obtaining emotional first aid, and (6) moving on [6]. Comparing these stages with the second victims’ response process identified in this study, the response stages were considered to be similar, overall. In particular, in the present study, it was highlighted that the memories of the PSIs faded but were not erased completely, remaining for prolonged periods. The final stage of recovery of second victims proposed by Scott et al. [6] is divided into three paths: dropping out, surviving, and thriving. In each path, it can be seen that the event continues to affect the second victims, although there may be a difference of degree of impact. Therefore, a support program for second victims would require teaching victims to positively sublimate such memories and learn to accept the incident, rather than taking an approach to encouraging healthcare professionals to repress memories of PSIs. This is the reason the term “response process” is used in this study rather than the term “recovery process.” In some cases, the second victims took passive countermeasures, like avoiding similar situations, but some engaged in active coping to prevent recurrence of the case. Thus, achieving recovery through resilience seems to be an appropriate approach, and support that helps second victims transcend their trauma and transform their experience into a positive memory is suggested.

At present, there are a number of efforts underway to develop and apply programs to help second victims overcome their emotional difficulties, especially in the United States [18–22]; and like these programs, it is necessary to introduce a program suited to the Korean situation, such as providing tailored support for the victims’ response. The results of the present study show that it is essential to gain an understanding of the necessary response steps for second victims and provide emotional or administrative support accordingly. Further, instilling an understanding of the general response process in second victims can also be helpful as they endeavor to surmount their emotional difficulties. It is important to quantify the degree of difficulty second victims are experiencing, such as by using a rating scale, and provide support based on the severity of the symptoms [23], while also providing emotional and administrative support based on each individual’s own response process. Such efforts can be expected to increase the effectiveness of a support program for second victims.

PSIs can cause a range of symptoms in second victims, and it is noteworthy that the responses of patients, caregivers, and fellow healthcare professionals influence second victims’ acceptance of and responses to the incident. Particularly, in some cases, fellow healthcare professionals accepted the participants’ mistakes and empathized with them, while others reacted angrily or criticized the participants in public. In addition, the atmosphere in the medical institutions regarding discussing PSIs and the institutions’ standards of patient safety culture affected the response process. Thus, the patient safety culture in medical institutions can have an important influence on the recovery of second victims. It is consequently important to establish in every medical institution, programs that focus on patient safety education and raising the awareness of support program for second victims [13].

It was also confirmed in this study that not only support for overcoming the psychological difficulties experienced by second victims, but also practical administrative support, such as addressing legal problems, should be provided. The participants were unaware of how to express their feelings of remorse and respond after an incident. Moreover, they emphasized the necessity of administrative support that could help them discuss or systematically address possible legal proceedings against them [24]. Thus, it is crucial to assist healthcare professionals in managing the disclosure of PSIs, which concerns communicating with patients and caregivers after a PSI. As disclosure of PSIs is known to reduce healthcare professionals’ feelings of guilt, it is expected that such a measure can improve the relationship between patients and caregivers and healthcare professionals, while also alleviating the symptoms of second victims [25, 26]. In other words, coaching healthcare professionals regarding the disclosure of PSIs must be considered a prerequisite for a support program for second victims [27].

The majority of the literature referring to the second victim issue has been published in western countries such as the United States, so it was necessary to ascertain whether there are cultural differences in this regard. However, as verified in this study, eastern-based (i.e., in Korea) second victims who experienced a PSI also encounter various emotional reactions such as embarrassment, guilt, and depression, as well as behavioral changes such as insomnia and avoidance.^4^ This research, however, also revealed that the effects of factors other than incident, such as work-related factors, could affect second victims’ responses. For instance, many participants commented that they did not have sufficient time to think about the case itself or to address patients and caregivers due to their heavy workload. In other words, work intensity and conditions in Korea influence the patient safety issue, and consequently also impact the second victims [28].

This study has several limitations. First, we derived the results of the study with a small sample and participants from tertiary hospitals. Therefore, further studies are needed, such as those with more participants using the survey method and studies on participants working in other types of medical institutions. In particular, it will be meaningful to assess the level of psychological impact due to PSIs using measures such as Impact of Event Scale [29]. Second, the possibility that the participants’ responses were biased by knowledge of the researchers’ intention cannot be completely excluded. Thus, we tried to ensure the validity of the study by cross-checking the participants’ responses and conducting a mutual review among the researchers as mentioned in the method, especially in the research validity assessment section. Third, the participants’ opinions regarding the specific content that should be included in a second victim support program were not explored in this study. Although the participants in this study mentioned that emotional and administrative support was necessary to address the second victim issue, they were not specifically asked for their impressions on the support programs for second victims currently being developed and implemented in other countries. It is recommended that in future research, a survey concerning the content of such support programs for second victims be conducted. Fourth, the severity of the second victims’ symptoms was not investigated. In future studies, it would be meaningful to quantify the extent of the difficulties experienced by second victims [23, 29] and ascertain whether there is a difference in experience or opinion depending on the severity of such symptoms.

## Conclusions

In conclusions, this research is significant because it provides a comprehensive understanding of second victims’ experiences in an eastern region, Korea, obtaining this information using a qualitative research method. The findings of the study also highlight the five stages of the second victim response process, and it can be used to design a specialized second victim support program in Korea. This research could also be utilized as a basis for the development and implementation of an effect evaluation tool for second victim support programs.

## Additional file

Additional file 1: Interview guideline. This is the semi-structured interview guideline used in interviews. (DOCX 19 kb)

## Abbreviations

PSI: Patient safety incidents
