# Supplementary material for: Experiences and responses of second victims of patient safety incidents in Korea: a qualitative study
Source: BMC Health Serv Res. 2019 Feb 6;19:100. doi: 10.1186/s12913-019-3936-1 (PMC6366082; doi:10.1186/s12913-019-3936-1)
Supplement: Supplementary file 1 — Interview guideline. This is the semi-structured interview guideline used in interviews. (DOCX 19 kb) [file 12913_2019_3936_MOESM1_ESM.docx]

Additional file 1: Interview guideline.

[Introduction]

1. Introduction to the interview

- Introduction to the in-depth interview and explanation of the purpose, etc.
- Guidance on the in-depth interview progress

2. Information about the protection of privacy

3. Collection of participants’ information such as occupation, department, and work experience

[Experiences of patient safety incidents]

1. Could you tell me about your patient safety incidents?

- If it is difficult for the participant to narrate the incident, interviewer prompts the participant to recall the surrounding at the time of the incident.

2. You would have had a hard time. How did you feel at that time?

- Identified major emotional responses

3. Did you get physically tired due to the incident at that time? For example, did you have insomnia?

- Identified major physical responses

4. Do you think that you, especially your work, were hindered by the incident? How was it?

- Identified major behavioral responses

[Deal with the patient safety incidents]

1. How was the patient safety incident resolved?

2. How have you been affected by the patient safety incident? Have you made any changes in your medical practice?

- If there was a change, what do you think was the reason?

3. Did you talk to a colleague or senior about the patient safety incident?

- What was the response of your colleagues or seniors?
- Did you report to the patient safety reporting system?
- Did you talk to your family about the occurrence of the patient safety incident and handling the incident?

4. How did you respond to the patient or caregiver involved in the patient safety incident?

- Identified whether participant had engaged in disclosure
- What if you did disclosure of patient safety incident?

5. How did you resolve your feelings?

- Identified countermeasures such as denial, discounting, distancing, etc.

[Need for support]

1. Did the hospital support you in resolving the patient safety incident?

- Did you feel hurt during the investigation of the patient safety incident?
- If you ever received counseling, did it help you get back to your daily activities?
- What about assigning a dedicated team or staff to handle patient safety incident?

2. What was the reaction or attitude of a colleague or senior to the patient safety incident?

- How did you want them to react?

3. What was the reaction or attitude of a family or friend to the patient safety incident?

- How did you want them to react?

[Finishing the discussions]

1. Let’s summarize some of the key points from interview. Is there anything else?

2. Do you have any questions?

3. Token of thanks for participating
